# Defect-Targeted Repair for Efficient and Stable Perovskite Solar Cells Using 2-Chlorocinnamic Acid

**DOI:** 10.3390/nano15161229

**Published:** 2025-08-12

**Authors:** Zhichun Yang, Mengyu Li, Jinyan Chen, Waqar Ahmad, Guofeng Zhang, Chengbing Qin, Liantuan Xiao, Suotang Jia

**Affiliations:** 1 State Key Laboratory of Quantum Optics Technologies and Devices, Institute of Laser Spectroscopy, Collaborative Innovation Center of Extreme Optics, Shanxi University, Taiyuan 030006, China; 202322618017@email.sxu.edu.cn (M.L.); 202222603001@email.sxu.edu.cn (J.C.); guofeng.zhang@sxu.edu.cn (G.Z.); chbqin@sxu.edu.cn (C.Q.); xlt@sxu.edu.cn (L.X.); tjia@sxu.edu.cn (S.J.); 2 Department of Physics, Qilu Institute of Technology, Jinan 250200, China

**Keywords:** perovskite solar cells, additive engineering, defect passivation, stability

## Abstract

Metal halide perovskites have appeared as a promising semiconductor for high-efficiency and low-cost photovoltaic technologies. However, their performance and long-term stability are dramatically constrained by defects at the surface and grain boundaries of polycrystalline perovskite films formed during the processing. Herein, we propose a defect-targeted passivation strategy using 2-chlorocinnamic acid (2-Cl) to simultaneously enhance the efficiency and stability of perovskite solar cells (PSCs). The crystallization kinetics, film morphology, and optical and electronic properties of the used formamidinium–cesium lead halide (FA_0.85_Cs_0.15_Pb(I_0.95_Br_0.05_)_3_, FACs) absorber were modulated and systematically investigated by various characterizations. Mechanistically, the carbonyl group in 2-Cl coordinates with undercoordinated Pb^2+^ ions, while the chlorine atom forms Pb–Cl bonds, effectively passivating the surface and interfacial defects. The optimized FACs perovskite film was incorporated into inverted (p-i-n) PSCs with a typical architecture of ITO/NiO_x_/PTAA/Al_2_O_3_/FACs/PEAI/PCBM/BCP/Ag. The optimal device delivers a champion power conversion efficiency (PCE) of 22.58% with an open-circuit voltage of 1.14 V and a fill factor of 82.8%. Furthermore, the unencapsulated devices retain 90% of their initial efficiency after storage in ambient air for 30 days and 83% of their original PCE after stress under 1 sun illumination with maximum power point tracking at 50 °C in a N_2_ environment, demonstrating the practical potential of dual-site molecular passivation for durable perovskite photovoltaics.

## 1. Introduction

Over the past decade, metal halide perovskite solar cells (PSCs) have demonstrated unprecedented progress in the photovoltaic technology, achieving remarkable increase in power conversion efficiency (PCE) from 3.8% to 27.0% [1,2,3], comparable to that of the traditional single-crystal silicon solar cells [4,5,6,7,8,9,10]. This rapid advancement is ascribed to the exceptional optoelectronic properties of perovskites, including the high absorption coefficients [11,12,13], tunable bandgaps [14,15,16,17], and excellent charge carrier mobilities [18,19,20,21]. However, the commercialization of PSCs remains challenged by issues of long-term stability and performance scalability, primarily due to defects in polycrystalline perovskite films. These defects, preferentially distributed at the surface and/or grain boundaries (GBs), arise from uncontrollable factors during film formation and the inherent low formation energy of hybrid perovskites, making them vulnerable to moisture, oxygen, light, and thermal stresses.

Defects in perovskite films, such as undercoordinated Pb^2+^ ions, halide vacancy sites, and interstitials species, generally serve as non-radiative recombination centers. They not only decrease open-circuit voltage (*V*_OC_) and carrier lifetimes but also accelerate ion migration under illumination. This ion migration intensifies material decomposition, particularly at GBs [22,23,24,25], facilitating the moisture and oxygen permeation, which further degrade the film quality and device stability. These defect-related problems significantly limit the efficiency and durability of PSCs and highlight the urgent needs for innovative material engineering to mitigate their impact and advance this photovoltaic technology towards practical applications [26].

A wide range of approaches have been explored for developing effective defect mitigation, including surface passivation, electrode modification, encapsulation techniques, and additive engineering. Among these, additive engineering has emerged as particularly effective, as it allows the incorporation of functional molecules, including small organic molecules, Lewis acids/bases, inorganic salts, and polymers, into perovskite precursor solutions without disrupting the lattice structure. These additives reduce defect densities, improve crystal orientation, and facilitate efficient charge extraction [27,28,29]. For instance, Yang et al. demonstrated that amino (N-H) and carbonyl (C=O) groups of theophylline coordinate with Pb^2+^ and form hydrogen bonds with iodine, respectively, to attain optimal passivation when these groups are in conjugated positions [30]. Similarly, Cheng et al. developed a polypeptide that effectively lowers charge recombination by interacting with undercoordinated Pb^2+^ and I^−^ ions [31]. Hu et al. reported a chlorine-based passivation strategy that effectively passivates surface defects through strong Pb-Cl bonds without incorporating Cl into the perovskite lattice [32]. Liu et al. demonstrated that neostigmine methyl sulfate molecules delay crystallization of perovskites and passivate both positive and negative charge defects via C=O interactions with Pb^2+^ ions [33]. Although such approaches reduce defect densities and improve efficiency, they often rely on single-point passivation mechanisms and frequently fail to simultaneously optimize both film crystallinity and interface energetics.

Building on these developments, the present study proposes a novel defect-targeted passivation strategy that incorporates 2-chlorocinnamic acid (2-Cl), a small organic molecule with carbonyl and chloride functionalities, into the formamidinium–cesium lead halide (FA_0.85_Cs_0.15_Pb(I_0.95_Br_0.05_)_3_, FACs) perovskite precursor solution. As an electron donor, the carbonyl group in 2-Cl forms strong Pb=O bonds with undercoordinated Pb^2+^ ions, which improves the film crystallinity and lattice stability. Furthermore, the chloride ions in 2-Cl form Pb-Cl bonds, effectively passivating surface and GB defects. These interactions promote efficient charge carrier extraction, reduce density of defect states, and suppress non-radiative recombination. The effectiveness of this approach is demonstrated through comprehensive device performance evaluation. Leveraging this approach, our 2-Cl-modified device obtained a champion PCE of 22.58%, which is obviously higher than the maximum efficiency of 20.24% of the reference device. Additionally, the fill factor (FF) increased notably from 78.9% to 82.8%, reflecting an enhanced charge carrier transport and decreased recombination losses. In terms of stability, the modified devices retain 90% of their initial efficiency after storage in ambient air for 30 days and 83% of the original efficiency after stress under continuous 1 sun illumination with maximum power point tracking (MPPT) at 50 °C in a N_2_ atmosphere. These findings demonstrate the potential of 2-Cl as a flexible passivation agent to address both efficiency and stability challenges in PSCs. This study paves the way for stable and high-efficiency PSCs by improving defect management techniques.

## 2. Materials and Methods

Formamidinium iodide (FAI, 99.99%), lead (II) iodide (PbI_2_, 99.99%), nickel oxide particle (NiO_x_), phenethylammonium iodide (PEAI), and indium-doped tin oxide (ITO) glasses were purchased from Advanced Electronic Technology Co., Ltd., Shenzhen, China. N,N-dimethylformamide (DMF, 99.8%), dimethyl sulfoxide (DMSO, 99.7%), ethyl acetate (EA, 99.5%), isopropanol (IPA, 99.5%), and chlorobenzene (CB, 99.8%) were purchased from J&K, Shanghai, China. Poly[bis(4-phenyl) (2,4,6-trimethylphenyl) amine] (PTAA), [6,6]-Phenyl-C61-butyric acid methyl ester (PCBM), and bathocuproine (BCP) were obtained from Xi’an Polymer Light Technology Corporation Xi’an, China. Toluene (≥99.5%) was purchased from Sinopharm Chemical Reagent Co., Ltd., Shanghai, China. Cesium bromide (CsBr, >99.0%) and 2-chlorocinnamic acid (2-Cl) were purchased from Tokyo Chemical Industry Co., Ltd., Tokyo, Japan.

ITO substrates were firstly scribed by a pulsed laser. The etched substrates were ultrasonically cleaned with detergent, deionized water, ethyl alcohol, acetone, and isopropanol for 15 min, respectively. The cleaned substrates were dried at 100 °C in oven, and treated with UV–ozone (UVO) for 15 min. NiO_x_ nanoparticles (10 mg mL^−1^ in deionized water) were spin-coated on ITO substrate at 2500 r.p.m. for 30 s and annealed at 150 °C for 30 min. PTAA (2 mg mL^−1^ in toluene) was spin-coated at 5000 r.p.m. for 30 s and annealed at 100 °C for 10 min. Perovskite solution was prepared by dissolving 691.5 mg PbI_2_, 219.3 mg FAI, 47.8 mg CsBr, and 1 mg 2-Cl in 1 mL mixed solvent of DMF and DMSO with a volume ratio of 4:1. The prepared perovskite solution with 2-Cl was spin-coated on NiO_x_/PTAA substrate at 5000 r.p.m. for 60 s, and the antisolvent of EA was timely dropped at 15 s and annealed at 110 °C for 15 min. PEAI dissolved in IPA at a concentration of 1 mg mL^−1^ was spin-coated on the surface of perovskite films at 6000 r.p.m. for 30 s and annealed at 110 °C for 10 min. PCBM (20 mg mL^−1^ in CB) was spin-coated on perovskite film at 3000 r.p.m. for 30 s and then annealed at 70 °C for 10 min. The saturated BCP solution in IPA was then spin-coated at 6000 r.p.m. for 30 s. Finally, 100 nm of silver electrode was thermally evaporated. The active area of PSCs was 0.09 cm^2^.

The surface and cross-sectional images of perovskite films were observed by field-emission scanning electron microscopy (Hitachi SU 8010, Hitachi, Tokyo, Japan). The roughness of perovskite films was obtained by the atomic force microscopy (AFM, Bruker, Karlsruhe, Germany). X-ray diffraction (XRD) (D2 PHASER, Bruker, Karlsruhe, Germany) was carried out for the crystal structures of perovskite films. X-ray photoelectron spectroscopy (XPS, Thermo Fisher Scientific, Waltham, MA, USA) was conducted using a K-Alpha + spectrometer. Ultraviolet photoelectron spectroscopy (UPS, Thermo Fisher Scientific, Waltham, MA, USA) was carried out using the Escalab Xi+. Ultraviolet–visible (UV–Vis) (UNIC 3802, Shanghai, China), steady-state photoluminescence (PL, MS starter, Wenzhou, China), and time-resolved PL (TRPL) were performed to verify the optical quality of perovskite films. Fourier transform infrared (FTIR) was performed by the iS50 FT-IR (Nicolet, Jackson, WI, USA). Electrochemical impedance spectroscopy (EIS) and Mott–Schottky measurements were performed using an electrochemical workstation (CHI 760E, Shanghai Chenhua Instrument, Shanghai, China). The transient photovoltage (TPV) decay was determined using a homemade transient photoelectric test system. Transient absorption (TA, TIME-TECH SPECTRA, Dalian, China) spectroscopy was carried out to investigate the carrier dynamics. The dark current density and space charge limited current (SCLC) results were collected by a highly accurate source meter (2602B, Keithley, Solon, OH, USA). *J–V* curves of the fabricated devices were measured in ambient air by a Keithley 2400 source meter and solar simulator (SCX-100A, Beijing Zhongke Shicheng Technology Co., Ltd., Beijing, China). External quantum efficiency (EQE) results were obtained from a commercial system (FineDet 900, Oriental Spectra Technology (Guangzhou) Co., Ltd., Guangzhou, China).

## 3. Results

**Figure 1:**
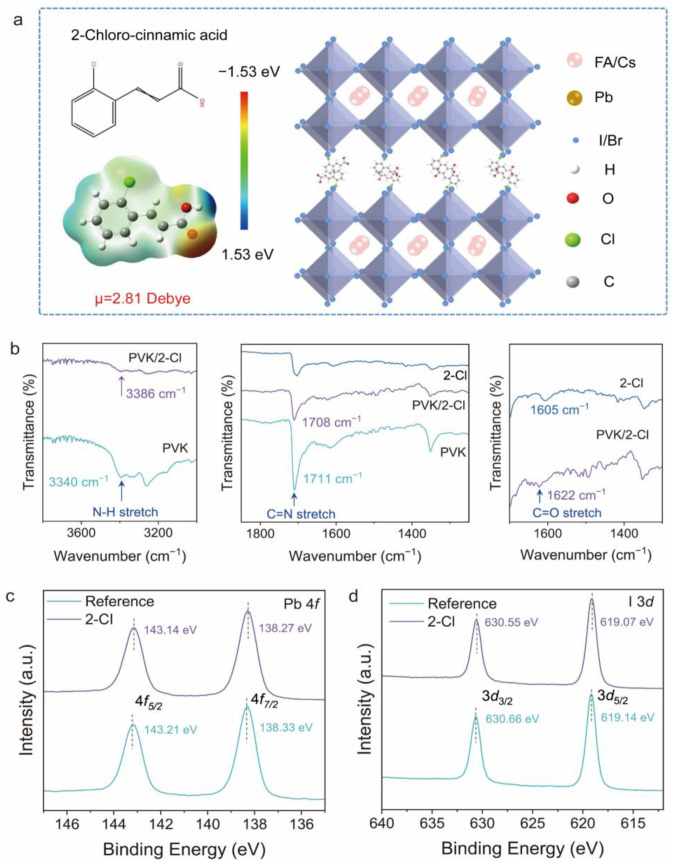
(**a**) The molecular structure and electrostatic potential (ESP) maps of 2-Cl. (**b**) The Fourier infrared spectroscopy of the reference perovskite (PVK), 2-Cl passivated perovskite, and pure 2-Cl powders. XPS of (**c**) Pb 4*f*, and (**d**) I 3*d* of perovskite films with and without 2-Cl modification.

Figure 1a and Figure S1 present the chemical structure and dipole moment of 2-Cl, with a Gaussian calculated value of 2.81 Debye. It can be observed that 2-Cl possesses both electron-rich and electron-deficient regions, indicating its potential to passivate a variety of charge defects. Specifically, the carbonyl group (C=O) at the terminal position of the 2-Cl molecule serves as an electron donor, providing lone electron pairs to coordinate with undercoordinated Pb^2+^ ions and form Pb=O bonds. Meanwhile, the chlorine atom on the benzene ring can form a Pb-Cl bond with the perovskite framework, which is stronger than the typical Pb-I bond, thereby further enhancing the passivation effect.

To further explore the interaction mechanisms between 2-Cl and perovskite precursors, Fourier transform infrared spectroscopy (FTIR) and X-ray photoelectron spectroscopy (XPS) were carried out. As shown in Figure 1b, the N-H stretching vibration peak of the original perovskite film appears at approximately 3340 cm^−1^. Upon the addition of 2-Cl molecule, this peak shift to 3386 cm^−1^. The observed peak shift and tensile vibration may result from the interaction between the perovskite and the additive molecule, specifically the formation of hydrogen bonds between 2-Cl and the FA^+^ cation. In addition, the perovskite powder containing 2-Cl exhibited a strong vibrational peak at 1622 cm^−1^, whereas the pure additive powder showed only a weak vibration at 1605 cm^−1^. Based on the FTIR spectra of the 2-Cl molecule, this shift is likely attributed to the stretching vibration of the C=O bond [34], suggesting that the additive interacts with the perovskites. The synergistic effect of hydrogen and coordination bonding interactions not only enhance the structural stability of the perovskite crystal lattice but also improve charge carrier dynamics and the overall performance of the PSCs [35]. XPS analysis further confirms this interaction and reveals the shift in the Pb 4*f* peaks (Figure 1c), indicating the formation of coordination between the C=O group in 2-Cl and undercoordinated Pb^2+^ ions in the perovskite matrix. The characteristic N 1*s* peak at 400.43 eV (Figure S2), originates from the FA^+^ cation in the pristine perovskite, confirms its existence in the lattice. In the presence of 2-Cl, the XPS spectra reveal a binding energy shift in I 3*d* of approximately 0.11 eV and 0.10 eV for N 1*s* (Figure 1d), respectively. These shifts suggest the formation of hydrogen bonds (C=O⋯H-N) between FA^+^ and the carbonyl group in 2-Cl. This interaction increases the electron density of the nitrogen atoms of FA^+^, resulting in the observed N 1*s* binding energy reduction. The synergistic interactions of 2-Cl with FA^+^, I^−^, and Pb^2+^ ions are beneficial for regulating the crystallization kinetics of perovskite, suppressing ion migration, and reducing defect formation, thereby enhancing perovskite film quality and device performance [36].

**Figure 2:**
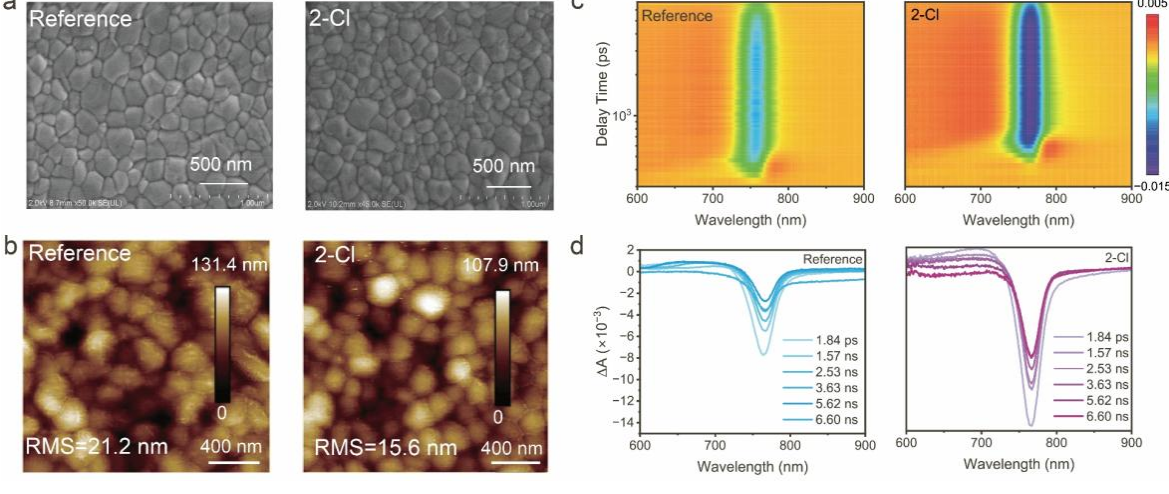
(**a**) Top-view SEM, and (**b**) AFM images of perovskite films without (Reference) and with 2-Cl. (**c**) 2D pseudo-color plots of fs-TA for the reference and 2-Cl-modified perovskite films, and (**d**) corresponding TA spectra at different decay times.

The effect of 2-Cl on the morphology of perovskite films was further investigated using scanning electron microscopy (SEM) and atomic force microscopy (AFM). As shown in Figure 2a, the 2-Cl-modified perovskite film exhibits a compact surface with a larger grain size (Figure S3). Notably, the film surface roughness decreased from 21.2 nm to 15.6 nm upon the incorporation of 2-Cl (Figure 2b), which is beneficial for improving the interfacial contact between the perovskite and electron transport layer (ETL) [37]. Moreover, ultraviolet photoelectron spectroscopy (UPS) was conducted to examine the influence of 2-Cl on the energy levels of perovskite film surfaces. Figure S4 shows the UPS spectra, including secondary electron cutoff valence band regions. From the cutoff edge, the work function and the valence band maximum (VBM) position were extracted, with detailed results presented in Supplementary Figure S5 and Table S1. The 2-Cl-modified film results reveal a slight reduction in work function and a downward shift in the valence band position. This adjustment facilitates the energy level alignment with the ETL, thereby promoting more efficient charge extraction and transport [38].

To further understand the carrier dynamics, femtosecond transient absorption (fs-TA) was employed, using 515 nm pump light to excite the sample surfaces. Two-dimensional (2D) pseudo-color maps of TA spectra for both reference and 2-Cl-modified perovskite films are presented in Figure 2c. Both films reveal a prominent ground-state bleaching (GSB) signal at ~760 nm, demonstrating strong photoinduced carrier generation. Notably, the 2-Cl-modified film exhibits a stronger bleaching signal, suggesting a higher density of photogenerated carriers. As shown in Figure 2d, the TA spectra at various delay times for the 2-Cl-modified film exhibit a slower decay and extended relaxation time, indicating suppressed trap-assisted non-radiative recombination. This observation is further supported by the carrier lifetime analysis shown in Figure S6, which confirms an extended carrier lifetime in the modified perovskite films, contributing more efficient electron extraction at the perovskite/ETL interface [39,40,41].

**Figure 3:**
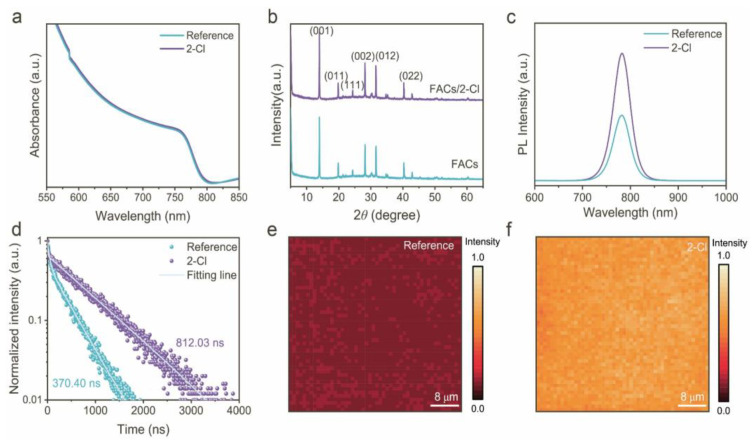
(**a**) UV–Vis absorption spectra of the reference and 2-Cl regulated perovskite films. (**b**) XRD patterns of the perovskite films without and with 2-Cl additives. (**c**) Steady-state PL spectra, and (**d**) TRPL spectra of the reference and 2-Cl-treated perovskite films. Normalized TRPL mapping of (**e**) reference and (**f**) 2-Cl-based perovskite films.

To investigate the optoelectronic properties, UV–Vis absorption spectra of both perovskite films were tested. As depicted in Figure 3a, both films exhibit similar absorption profiles with an optical cutoff edge around 780 nm. The optical bandgaps, derived from Tauc plots (Figure S7), were 1.58 eV for the reference sample and 1.57 eV for the 2-Cl-modified film, indicating that 2-Cl passivation has a negligible effect on the intrinsic bandgap of the perovskite film. To assess the influence of 2-Cl on the crystallization dynamics of perovskite films, in situ absorption spectra and XRD analysis were comparatively performed. The UV–Vis intensity was recorded during the spin-coating process with the assistance of an antisolvent. It is obvious that the crystallization was delayed (from 20 s to 23 s) with the introduction of the 2-Cl molecule (Figure S8). As shown in Figure 3b, both the reference and 2-Cl-modified films show strong diffraction peaks at 13.98°, 19.8°, 24.4°, 28.2°, 31.6°, and 40.4°, corresponding to the (001), (011), (111), (002), (012), and (022) crystal planes of the perovskite lattice, respectively [42]. Notably, the full width at half maximum (FWHM) of the (001) peak at 13.98° is reduced from 0.149 (reference) to 0.141 (2-Cl regulated film), as shown in Figure S9, indicating improved crystallinity upon 2-Cl incorporation.

Furthermore, the impact of 2-Cl on carrier dynamics was assessed via steady-state photoluminescence (PL) and time-resolved photoluminescence (TRPL) spectroscopy, as presented in Figure 3c,d. The 2-Cl-modified perovskite film exhibits significantly enhanced PL intensity compared to that of the reference film, with emission peaks at approximately 780 nm. This enhanced PL intensity suggests a reduced density of trap states and suppressed non-radiative recombination, demonstrating effective passivation of defect sites by 2-Cl. TRPL measurements were conducted to further investigate the carrier recombination kinetics. The decay curves were fitted using the following biexponential formula:

(1) $$Y=A_{1}\exp\left(\frac{-t}{\tau_{1}}\right)+A_{2}exp\left(\frac{-t}{\tau_{2}}\right)+y_{0}$$

where $A_{1}$ and $A_{2}$ are the relative amplitudes, while $\tau_{1}$ and $\tau_{2}$ are the lifetimes for the fast and slow recombination, respectively. The average lifetimes ($\tau_{ave}$) of perovskite films are calculated by the following formula:

(2) $$\tau_{ave}=\frac{A_{1}\tau_{1}^{2}+A_{2}\tau_{2}^{2}}{A_{1}\tau_{1}+A_{2}\tau_{2}}$$

The TRPL results in Figure 3d and Table S2 demonstrate that 2-Cl-modified films display a longer carrier lifetime (812.03 ns) compared to the reference sample (370.40 ns), confirming a suppressed defect-assisted recombination pathways and improved charge retention within perovskite film [43]. Moreover, in order to verify the above conclusion, TRPL mapping was also performed to evaluate the carrier lifetime and homogeneity of the thin films, as shown in Figure 3e,f. The treated perovskite film has a stronger fluorescence lifetime and a more uniform distribution than the untreated film, indicating that dopants can passivate surface/interface defects. The 2-Cl-modified perovskite film exhibits a longer and more uniformly distributed PL signal compared to the reference film, indicating an enhanced film uniformity and a reduced non-radiative recombination. These findings confirm that the incorporation of 2-Cl successfully passivates surface and/or interfacial defects [44], thereby enhancing the optoelectronic properties of perovskite film.

**Figure 4:**
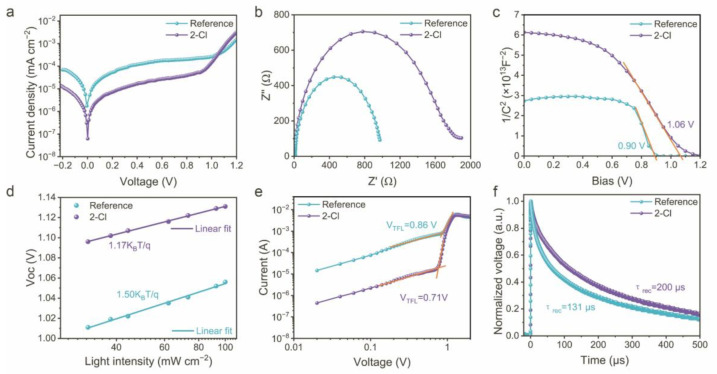
(**a**) *J–V* curves in dark conditions, (**b**) EIS, (**c**) Mott–Schottky plots, (**d**) light intensity dependence on *V*_OC_ plots, (**e**) SCLC, and (**f**) normalized TPV decay of for the devices without and with 2-Cl doping.

To determine the optimal doping concentration of 2-Cl in perovskite precursor ink, we fabricated devices incorporating 0.5, 1.0, 1.5, and 2.0 mg mL^−1^ of 2-Cl, along with the reference device without the additive. As evidenced by the photovoltaic parameters presented in Figure S10, the device with 1.0 mg mL^−1^ 2-Cl exhibited the best performance, confirming it as the optimal concentration. However, higher concentrations led to a gradual reduction in device performance parameters, which we attribute to increased trap density. This phenomenon causes more charge carriers to be trapped at grain boundaries, impeding charge separation into free electrons and holes, and compromising the photovoltaic performance. The champion and average photovoltaic parameters of all devices with different 2-Cl concentrations are summarized in Table S3. Additionally, *J–V* curves of the most efficient devices under both forward and reverse scanning conditions at different 2-Cl concentrations are shown in Figure S11. To further evaluate the passivation effect, the dark current of the devices with a representative architecture of ITO/NiO_x_/PTAA/Al_2_O_3_/FACs perovskite/PEAI/PCBM/BCP/Ag was measured. As shown in Figure 4a, the 2-Cl-modified devices reveal lower leakage currents compared to the reference sample, correlating with the improved FF [37]. Electrochemical impedance spectroscopy (EIS) results and relevant fitting data are shown in Figure 4b and Table S4, respectively. These results further confirmed higher recombination resistance and smaller charge carrier transport resistance in 2-Cl-modified devices, aligning with prior studies showing suppressed non-radiative recombination and higher carrier transport. These findings are consistent with the TRPL measurements, which revealed prolonged carrier lifetimes observed in the 2-Cl-modified perovskite film. Furthermore, Mott–Schottky analysis (Figure 4c) reveals a higher flat-band potential of 1.06 V for the 2-Cl-modified device, compared to 0.90 V for the reference device, representing a stronger built-in electric field, which facilitates the charge separation, as supported by the following equation:

(3) $$\frac{1}{C^{2}}=\frac{2(V_{bi}-V)}{q\varepsilon \varepsilon_{0}A^{2}N}$$

where *C* is the depletion layer capacitance, *V*_bi_ is the built-in potential, *q* is the elementary charge, *ε* is the perovskite dielectric constant, *ε*_0_ is the vacuum dielectric constant, A is the effective area of the device, and *N* is the carrier concentration [45,46]. Additionally, Figure 4d illustrates the variation of *V*_OC_ with light intensity, where the plot slope offers insights into recombination mechanisms. The linear logarithmic relationship between *V*_OC_ and light intensity is observed, as in the following expression:

(4) $$V_{OC}=\left(\frac{nk_{B}T}{q}\right)\;ln\;(\frac{J_{ph}}{J_{0}}+1)$$

where *n* is the ideality factor, *k*_B_ is the Boltzmann constant, *T* is the categorical temperature, *q* is the elementary charge, *J*_ph_ is the photocurrent density, and *J*_0_ is the reverse saturation current density. An *n* value approaching 1 indicates dominant bimolecular radiative recombination, while larger values suggest significant trap-assisted non-radiative recombination. The reduced *n* value (1.17) of the 2-Cl-modified device implies fewer trap-induced recombination as compared to the reference device (1.50). This reduction in *n* revealed that incorporation of 2-Cl additive contributes to defect passivation and improves the overall device performance [47].

To further investigate the origins of changes in built-in potential, space-charge-limited current (SCLC) measurements were conducted using an electron-only device with an ITO/SnO_2_/perovskite/PCBM/Ag architecture (Figure S13). The *I–V* characteristics under dark conditions were used to determine the trap density (*N*_trap_) of perovskite films. As shown in Figure 4e, the current increases linearly with low voltage, characteristics of the ohmic region. Upon exceeding a threshold voltage, a rapid nonlinear increase indicates the trap-filled limit region. The transition point between these regions is defined as the trap-filled limit voltage (*V*_TFL_). The measured *V*_TFL_ values for the reference and 2-Cl-modified devices are 0.86 V and 0.71 V, respectively, indicating significant differences in defect populations. The corresponding trap density was calculated using the following equation:

(5) $$N_{trap}=\frac{2\varepsilon_{0}\varepsilon V_{TFL}}{qL^{2}}$$

where *q* is the elementary charge, *L* is the film thickness of the perovskite film (~505 nm, as determined from the cross-sectional SEM in Figure S12), ε_0_ is the vacuum permittivity, and *ε* is the relative dielectric constant of FACs perovskite (~24.2) [48,49]. The calculated *N*_trap_ values for the reference and 2-Cl-modified devices are found to be 9.03 × 10^15^ cm^−3^ and 7.46 × 10^15^ cm^−3^, respectively, demonstrating a 17.4% reduction in trap density through 2-Cl passivation. These findings correlate well with the *V*_OC_ enhancement discussed earlier. The effective defect passivation by 2-Cl reduces carrier recombination and facilitates charge transport. To confirm this trend, we further performed the complementary trap density measurements using hole-only devices (ITO/NiO_x_/FACs perovskite/PTAA/Ag) under dark conditions (Figure S14). Additionally, transient photovoltage (TPV) measurements provided additional evidence to assess carrier dynamics. As shown in Figure 4f, the carrier lifetime of the 2-Cl-modified device was 200 μs, higher than the 131 μs observed in the reference device. This result further confirms the suppression of non-radiative recombination and promotes carrier transport due to 2-Cl doping. These findings align with the TA measurements, reinforcing the effectiveness of 2-Cl in passivating defects and enhancing PSC performance.

**Figure 5:**
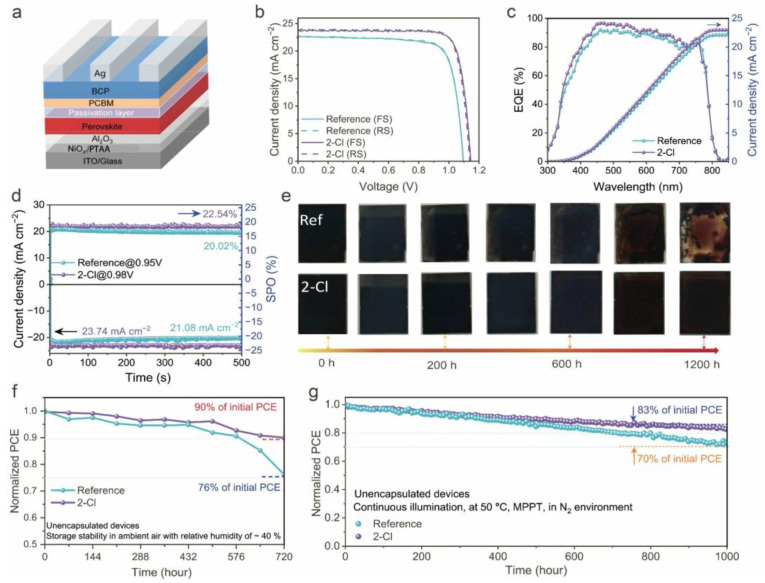
(**a**) Structure illustration of the fabricated PSCs. (**b**) *J–V* curves of the champion efficiency. (**c**) EQE spectra and the integrated current density for the 2-Cl-doped and reference devices. (**d**) Steady-state power and photocurrent density output at the maximum power point. (**e**) Photographs of the reference and 2-Cl treated perovskite films aging in ambient air with RH of ~40%. (**f**) Long-term storage stability of the unencapsulated devices in ambient air with a relative humidity of ~40%. (**g**) Operational stability of the unencapsulated devices under continuous 1 sun equivalent illumination at 50 °C with MPPT in a N_2_ atmosphere.

To verify the effectiveness of 2-Cl in improving device performance, p-i-n devices with the structure ITO/NiO_x_/PTAA/Al_2_O_3_/FACs perovskite/PEAI/PCBM/BCP/Ag were fabricated, as shown in Figure 5a. *J–V* curves for both the champion reference and 2-Cl-modified devices are displayed in Figure 5b. The optimal 2-Cl-modified device achieved a PCE of 22.58%. The photovoltaic parameters of the most efficient reference device and modified target device are summarized in Figure S15 and Table S5. The external quantum efficiency (EQE) spectra and integrated short-circuit current density (*J*_SC_) (Figure 5c) of both devices align with the *J–V* measurements, showing a deviation of less than 5% [38]. The steady-state power output measurements (Figure 5d) show that the 2-Cl-modified device achieves a stable PCE of 22.54%, compared to 20.02% for the reference sample, confirming the reliability of the high-performance results. To evaluate environmental stability, both the reference and 2-Cl-modified perovskite films were exposed to ambient air (RH ≈ 40%, 25 °C). As shown in Figure 5e, the reference film exhibited edge decomposition after 200 h and was nearly fully decomposed after 1200 h, whereas the 2-Cl-modified film exhibited only partial degradation after 1200 h [50,51]. This enhanced stability is attributed to the higher water contact angle of the 2-Cl-modified film, as shown in Figure S16. Additionally, the storage stability of unencapsulated 2-Cl-modified devices retained 90% of its initial PCE (Figure 5f) under ~40% RH over 720 h, outperforming the reference device. Moreover, the 2-Cl-modified device demonstrates an improved storage stability in a N_2_ atmosphere (Figure S17). The thermal stability tests (Figure S18) further confirm the enhanced durability of the 2-Cl-modified device. Operating stability was also examined under continuous 1 sun illumination with MPPT at 50 °C in a N_2_ environment. The PCE of the reference device decreased rapidly, retaining only 70% of its initial performance after 1000 h, whereas the 2-Cl-modified device retained 83% (Figure 5g). These enhanced environmental and operational stability are attributed to the improved perovskite film quality induced by 2-Cl, enhanced crystallinity, reduced defect density, and optimized carrier dynamics, consistent with prior analyses.

## 4. Conclusions

This study highlights the effectiveness of 2-Cl as a multifunctional additive for improving the stability and performance of PSCs. Incorporating 2-Cl into the perovskite precursor solution results in significant improvements in film quality and device performance. The incorporated 2-Cl facilitates the regulation of crystallization dynamics of perovskites and larger grain sizes. The carbonyl (C=O) group in 2-Cl coordinates with the undercoordinated Pb^2+^ ions and successfully passivates defects at grain boundaries and/or surfaces, suppressing non-radiative recombination. Concurrently, the combined effects of hydrogen bonding and coordination interactions improve the structural stability of the perovskite lattice, which improves charge carrier transport and extraction. As a result, the optimized device obtained a champion power conversion efficiency of 22.58%, with an impressive open-circuit voltage of 1.14 V and a fill factor of 82.8%. Furthermore, the devices displayed outstanding long-term stability, retaining 90% of their initial PCE after 30 days of ambient air exposure and 83% after 1000 h of MPPT at 50 °C in nitrogen environment. These findings establish 2-Cl as a promising additive for regulating crystallization, defect passivation, and enhancing device performance and durability, offering an effective strategy for advancing efficient and stable PSCs.

## Data Availability

Data supporting the findings are contained within the article and the Supplementary Materials.

## Supplementary Materials

The following supporting information can be downloaded at: https://www.mdpi.com/article/10.3390/nano15161229/s1, Figure S1: Dipole moments of the 2-Cl molecule; Figure S2: XPS spectra of the reference and 2-Cl-doped films; Figure S3: The grain size statistics of perovskite films; Figure S4: UPS analysis of perovskite films; Figure S5: Energy-level scheme for the reference and 2-Cl perovskite films based on the parameters derived from UPS spectra; Figure S6: The normalized fs-TA kinetics of perovskite films without and with 2-Cl addition; Figure S7: Tauc plots of perovskite films extracted from the UV–Vis absorption spectra; Figure S8: In situ UV–Vis absorption mapping of perovskite films; Figure S9: Full width at half maximum (FWHM) of (001) peaks calculated by the XRD results of the reference and 2-Cl perovskite films; Figure S10: The impact of 2-Cl concentration on device photovoltaic performance; Figure S11: *J*–*V* curves of the optimal devices doped with 2-Cl at various concentrations; Figure S12: Cross-sectional SEM image of the fabricated device with 2-Cl; Figure S13: Structure of the electron-only device used for SCLC measurements; Figure S14: Structure and results of the hole-only device used for SCLC measurements; Figure S15: Statistic photovoltaic parameters of PSCs based on the reference and 2-Cl-modified perovskite films; Figure S16: Contact angle results of perovskite films without (reference) and with 2-Cl; Figure S17: Storage stability of the fabricated devices in a N_2_-filled glovebox. Figure S18: Thermal stability of the reference and 2-Cl-modified devices; Table S1: Electronic parameters of the reference and 2-Cl passivated perovskite films derived from the UPS results; Table S2: Summary of TRPL lifetimes for the reference and 2-Cl-passivated perovskite films at a concentration of 1 mg mL^−1^; Table S3: Parameters of 2-Cl-doped devices at various concentrations; Table S4: Fitting parameters of impedance spectroscopy for the reference and 2-Cl-doped devices; Table S5: Photovoltaic parameters of the reference and 2-Cl-doped devices at a concentration of 1 mg mL^−1^.

## References

[1] Kojima A., Teshima K., Shirai Y., Miyasaka T. (2009). Organometal Halide Perovskites as Visible-Light Sensitizers for Photovoltaic Cells. J. Am. Chem. Soc..

[2] Tayari F., Teixeira S.S., Graca M.P.F., Nassar K.I. (2025). Progress and Developments in the Fabrication and Characterization of Metal Halide Perovskites for Photovoltaic Applications. Nanomaterials.

[3] Best Research-Cell Efficiency Chart. https://www.nrel.gov/pv/cell-efficiency.

[4] Jeong M., Choi I.W., Go E.M., Cho Y., Kim M., Lee B., Jeong S., Jo Y., Choi H.W., Lee J. (2020). Stable perovskite solar cells with efficiency exceeding 24.8% and 0.3-V voltage loss. Science.

[5] Zhang T., Wang F., Kim H.-B., Choi I.-W., Wang C., Cho E., Konefal R., Puttisong Y., Terado K., Kobera L. (2022). Ion-modulated radical doping of spiro-OMeTAD for more efficient and stable perovskite solar cells. Science.

[6] Zhao Y., Ma F., Qu Z., Yu S., Shen T., Deng H.-X., Chu X., Peng X., Yuan Y., Zhang X. (2022). Inactive (PbI_2_)_2_RbCl stabilizes perovskite films for efficient solar cells. Science.

[7] Li Z., Li B., Wu X., Sheppard S.A., Zhang S., Gao D., Long N.J., Zhu Z. (2022). Organometallic-functionalized interfaces for highly efficient inverted perovskite solar cells. Science.

[8] Jang Y.-W., Lee S., Yeom K.M., Jeong K., Choi K., Choi M., Noh J.H. (2021). Intact 2D/3D halide junction perovskite solar cells via solid-phase in-plane growth. Nat. Energy.

[9] Jiang Q., Zhao Y., Zhang X., Yang X., Chen Y., Chu Z., Ye Q., Li X., Yin Z., You J. (2019). Surface passivation of perovskite film for efficient solar cells. Nat. Photonics.

[10] Min H., Lee D.Y., Kim J., Kim G., Lee K.S., Kim J., Paik M.J., Kim Y.K., Kim K.S., Kim M.G. (2021). Perovskite solar cells with atomically coherent interlayers on SnO_2_ electrodes. Nature.

[11] Zhu L., Zhao R., Zhuang R., Wu T., Xie L., Hua Y. (2023). Slowing the hot-carrier cooling by an organic small molecule in perovskite solar cells. EcoMat.

[12] Tayari F., Teixeira S.S., Graca M.P.F., Nassar K.I. (2025). A Comprehensive Review of Recent Advances in Perovskite Materials: Electrical, Dielectric, and Magnetic Properties. Inorganics.

[13] Yin Z., Geng H., Yang P., Shi B., Fan C., Peng Q., Wu H., Jiang Z. (2021). Improved proton conduction of sulfonated poly (ether ether ketone) membrane by sulfonated covalent organic framework nanosheets. Int. J. Hydrogen Energy.

[14] Huang S., Huang P., Wang L., Han J., Chen Y., Zhong H. (2019). Halogenated-methylammonium based 3D halide perovskites. Adv. Mater..

[15] Ran C., Xu J., Gao W., Huang C., Dou S. (2018). Defects in metal triiodide perovskite materials towards high-performance solar cells: Origin, impact, characterization, and engineering. Chem. Soc. Rev..

[16] Li N., Tao S., Chen Y., Niu X., Onwudinanti C.K., Hu C., Qiu Z., Xu Z., Zheng G., Wang L. (2019). Cation and anion immobilization through chemical bonding enhancement with fluorides for stable halide perovskite solar cells. Nat. Energy.

[17] Yang L., Feng J., Liu Z., Duan Y., Zhan S., Yang S., He K., Li Y., Zhou Y., Yuan N. (2022). Record-Efficiency Flexible Perovskite Solar Cells Enabled by Multifunctional Organic Ions Interface Passivation. Adv. Mater..

[18] Degani M., An Q., Albaladejo-Siguan M., Hofstetter Y.J., Cho C., Paulus F., Grancini G., Vaynzof Y. (2021). 23.7% Efficient inverted perovskite solar cells by dual interfacial modification. Sci. Adv..

[19] Tayari F., Nassar K.I., Carvalho J.P., Teixeira S.S., Hammami I., Gavinho S.R., Graça M.P.F., Valente M.A. (2025). Sol–Gel Synthesis and Comprehensive Study of Structural, Electrical, and Magnetic Properties of BiBaO_3_ Perovskite. Gels.

[20] Chang X., Fang J., Fan Y., Luo T., Su H., Zhang Y., Lu J., Tsetseris L., Anthopoulos T.D., Liu S. (2020). Printable CsPbI_3_ Perovskite Solar Cells with PCE of 19% via an Additive Strategy. Adv. Mater..

[21] Zhu H., Liu Y., Eickemeyer F.T., Pan L., Ren D., Ruiz-Preciado M.A., Carlsen B., Yang B., Dong X., Wang Z. (2020). Tailored amphiphilic molecular mitigators for stable perovskite solar cells with 23.5% efficiency. Adv. Mater..

[22] Wang L., Chang B., Li H., Wu Y., Liu Z., Pan L., Yin L. (2022). [PbX_6_]^4−^ modulation and organic spacer construction for stable perovskite solar cells. Energy Environ. Sci..

[23] Huang S., Jiao M., Wang X., He X. (2022). A first-principles study on the structural and carrier transport properties of inorganic perovskite CsPbI_3_ under pressure. Crystals.

[24] Hassan A., Wang Z., Ahn Y.H., Azam M., Khan A.A., Farooq U., Zubair M., Cao Y. (2022). Recent defect passivation drifts and role of additive engineering in perovskite photovoltaics. Nano Energy.

[25] Chen J., He D., Park N.-G. (2022). Methodologies for >30% efficient perovskite solar cells via enhancement of voltage and fill factor. Sol. RRL.

[26] Guo Z., Jena A.K., Miyasaka T. (2022). Halide perovskites for indoor photovoltaics: The next possibility. ACS Energy Lett..

[27] Ren Y., Chen J., Ji D., Sun Y., Li C. (2020). Improve the quality of HC(NH_2_)_2_PbI_x_Br_3−x_ through iodine vacancy filling for stable mixed perovskite solar cells. Chem. Eng. J..

[28] Hu P., Huang S., Guo M., Li Y., Wei M. (2022). Ionic Liquid-Assisted Crystallization and Defect Passivation for Efficient Perovskite Solar Cells with Enhanced Open-Circuit Voltage. ChemSusChem.

[29] Zhu J., Hu X., Liu Z., Guo M., Zhang Y., Li Y., Li J., Wei M. (2023). UV-robust and efficient perovskite solar cells enabled by interfacial photocatalysis suppression and defect passivation. J. Mater. Chem. A.

[30] Wang R., Xue J., Wang K.-L., Wang Z.-K., Luo Y., Fenning D., Xu G., Nuryyeva S., Huang T., Zhao Y. (2019). Constructive molecular configurations for surface-defect passivation of perovskite photovoltaics. Science.

[31] Li M., Yue Z., Ye Z., Li H., Luo H., Yang Q.D., Zhou Y., Huo Y., Cheng Y. (2024). Improving the efficiency and stability of MAPbI_3_ perovskite solar cells by dipeptide molecules. Small.

[32] Wu J., Li M.-H., Fan J.-T., Li Z., Fan X.-H., Xue D.-J., Hu J.-S. (2023). Regioselective multisite atomic-chlorine passivation enables efficient and stable perovskite solar cells. J. Am. Chem. Soc..

[33] Ma X., Yang X., Wang M., Qin R., Xu D., Lan C., Zhao K., Liu Z., Yu B., Gou J. (2025). Comprehensive passivation on different charged ions and defects for high efficiency and stable perovskite solar cells. Adv. Energy Mater..

[34] Liu C., He B., Bao F., Cheng Q., Yang Z., Wei M., Ma Z., Chen H., Duan J., Tang Q. (2025). Influence of p-π conjugation in π-π stacking molecules on passivating defects for efficient and stable perovskite solar cells. J. Energy Chem..

[35] Zheng X., Li Z., Zhang Y., Chen M., Liu T., Xiao C., Gao D., Patel J.B., Kuciauskas D., Magomedov A. (2023). Co-deposition of hole-selective contact and absorber for improving the processability of perovskite solar cells. Nat. Energy.

[36] Zhang J., She Y., Zhu Y., Su H., Zheng X., Yao Y., Li D., Liu S. (2024). Enhancing Performance and Stability of Perovskite Solar Cells with a Novel Formamidine Group Additive. Small.

[37] Xu T., Xiang W., Yang J., Kubicki D.J., Tress W., Chen T., Fang Z., Liu Y., Liu S. (2023). Interface Modification for Efficient and Stable Inverted Inorganic Perovskite Solar Cells. Adv. Mater..

[38] Wu J., Zhu R., Li G., Zhang Z., Pascual J., Wu H., Aldamasy M.H., Wang L., Su Z., Turren-Cruz S.-H. (2024). Inhibiting Interfacial Nonradiative Recombination in Inverted Perovskite Solar Cells with a Multifunctional Molecule. Adv. Mater..

[39] Zhao R., Wu T., Hua Y., Wang Y. (2025). Improving performance of perovskite solar cells enabled by defects passivation and carrier transport dynamics regulation via organic additive. Chin. Chem. Lett..

[40] Liu S., Guan X., Xiao W., Chen R., Zhou J., Ren F., Wang J., Chen W., Li S., Qiu L. (2022). Effective Passivation with Size-Matched Alkyldiammonium Iodide for High-Performance Inverted Perovskite Solar Cells. Adv. Funct. Mater..

[41] Wu B., Ning W., Xu Q., Manjappa M., Feng M., Ye S., Fu J., Lie S., Yin T., Wang F. (2021). Strong self-trapping by deformation potential limits photovoltaic performance in bismuth double perovskite. Sci. Adv..

[42] Christians J.A., Miranda Herrera P.A., Kamat P.V. (2015). Transformation of the Excited State and Photovoltaic Efficiency of CH_3_NH_3_PbI_3_ Perovskite upon Controlled Exposure to Humidified Air. J. Am. Chem. Soc..

[43] Zheng G., Zhu C., Ma J., Zhang X., Tang G., Li R., Chen Y., Li L., Hu J., Hong J. (2018). Manipulation of facet orientation in hybrid perovskite polycrystalline films by cation cascade. Nat. Commun..

[44] Zhao C., Zhang Q., Lyu Y., Liu J., Shen F., Liu H., Kong H., Han H., Krishna A., Xu J. (2024). Regulating the Crystallization of FAPbI_3_-Based Perovskite with a Furan Substituted Ethylammonium Additive for Achieving Highly Efficient Solar Cells. Adv. Funct. Mater..

[45] Zhang J., Zheng X., Cui Q., Yao Y., Su H., She Y., Zhu Y., Li D., Liu S. (2024). Manipulating the Crystallization of Perovskite via Metal-Free DABCO-NH_4_Cl_3_ Addition for High Efficiency Solar Cells. Adv. Funct. Mater..

[46] Zhang Z., Zhu R., Tang Y., Su Z., Hu S., Zhang X., Zhang J., Zhao J., Xue Y., Gao X. (2024). Anchoring Charge Selective Self-Assembled Monolayers for Tin–Lead Perovskite Solar Cells. Adv. Mater..

[47] Luo Y., Liu K., Yang L., Feng W., Zheng L., Shen L., Jin Y., Fang Z., Song P., Tian W. (2023). Dissolved-Cl_2_ triggered redox reaction enables high-performance perovskite solar cells. Nat. Commun..

[48] Liu S., Chen R., Tian X., Yang Z., Zhou J., Ren F., Zhang S., Zhang Y., Guo M., Shen Y. (2022). Boost the efficiency of nickel oxide-based formamidinium-cesium perovskite solar cells to 21% by using coumarin 343 dye as defect passivator. Nano Energy.

[49] Bertoluzzi L., Boyd C.C., Rolston N., Xu J., Prasanna R., O’Regan B.C., McGehee M.D. (2020). Mobile Ion Concentration Measurement and Open-Access Band Diagram Simulation Platform for Halide Perovskite Solar Cells. Joule.

[50] Stoumpos C.C., Malliakas C.D., Kanatzidis M.G. (2013). Semiconducting Tin and Lead Iodide Perovskites with Organic Cations: Phase Transitions, High Mobilities, and Near-Infrared Photoluminescent Properties. Inorg. Chem..

[51] McMeekin D.P., Holzhey P., Fürer S.O., Harvey S.P., Schelhas L.T., Ball J.M., Mahesh S., Seo S., Hawkins N., Lu J. (2023). Intermediate-phase engineering via dimethylammonium cation additive for stable perovskite solar cells. Nat. Mater..

