# Supplementary material for: Defect-Targeted Repair for Efficient and Stable Perovskite Solar Cells Using 2-Chlorocinnamic Acid
Source: Nanomaterials (Basel). 2025 Aug 12;15(16):1229. doi: 10.3390/nano15161229 (PMC12388658; doi:10.3390/nano15161229)
Supplement: Supplementary file 1 [file nanomaterials-15-01229-s001.zip › nanomaterials-3799898-supplementary.pdf]

Supporting Information for

Defect-Targeted Repair for Efficient and Stable Perovskite Solar Cells Using 2-Chlorocinnamic Acid

Zhichun Yang [1,*], Mengyu Li [1], Jinyan Chen [1], Waqar Ahmad [2,*], Guofeng Zhang [1], Chengbing Qin [1], Liantuan Xiao [1], Suotang Jia [1]

[1] State Key Laboratory of Quantum Optics Technologies and Devices, Institute of Laser Spectroscopy, Collaborative Innovation Center of Extreme Optics, Shanxi University, Taiyuan 030006, China;

[2] Department of Physics, Qilu Institute of Technology, Jinan 250200, China;

*** Corresponding Author**

E-mail: yangzhichun@sxu.edu.cn (Z.Y.); waqar.ahmad@qlit.edu.cn (W.A.)

Supporting Figures

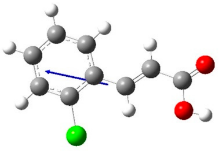

Figure S1. Dipole moments of the 2-Cl molecule.

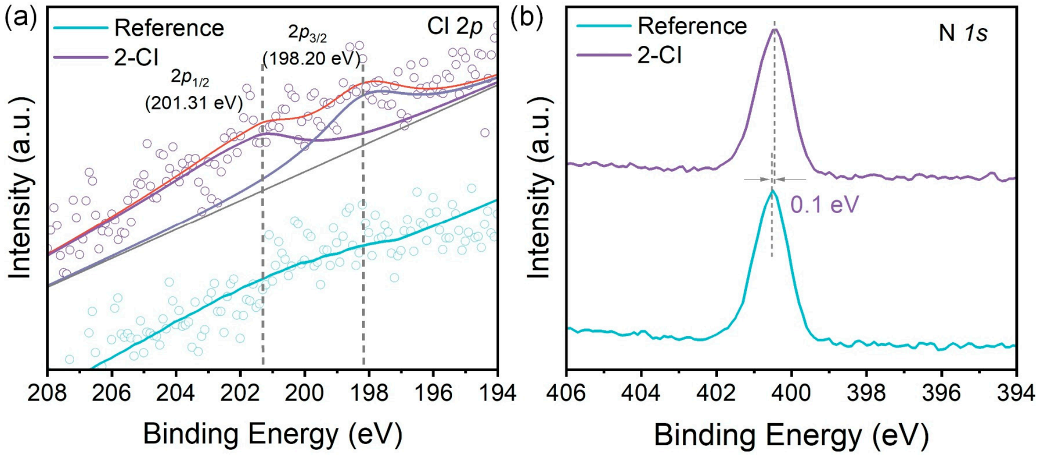

Figure S2. XPS spectra of the reference and 2-Cl-doped films. (a) Cl 2*p*, (b) N 1*s*.

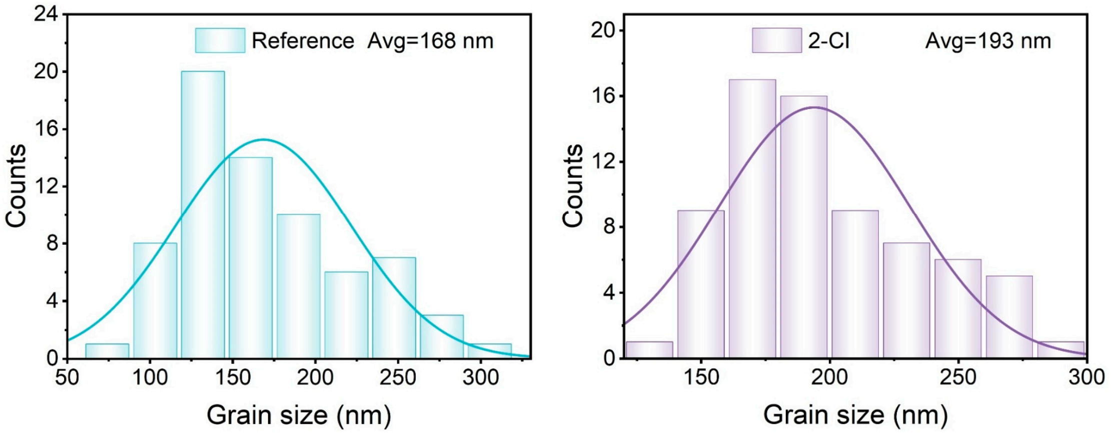

Figure S3. The grain size statistics of perovskite films.

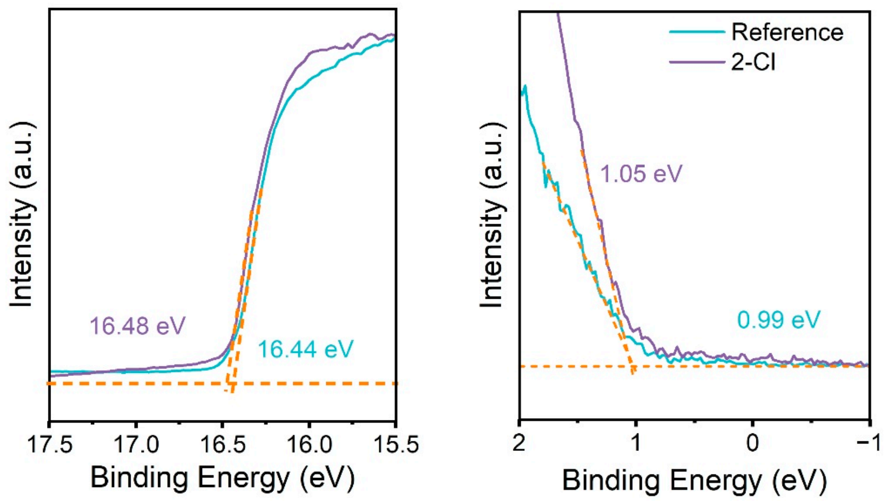

Figure S4. UPS analysis of perovskite films.

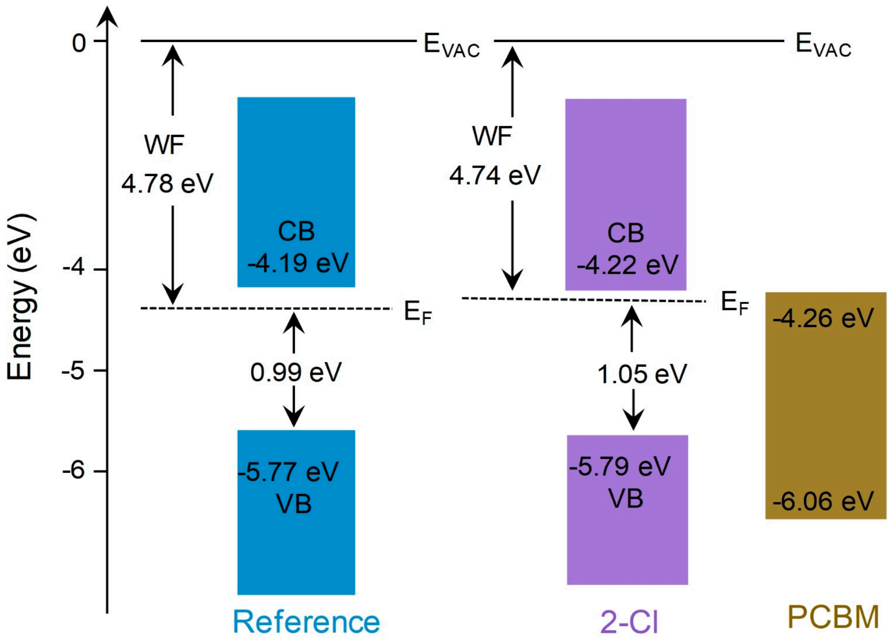

Figure S5. Energy-level scheme for the reference and 2-Cl perovskite films based on the parameters derived from UPS spectra.

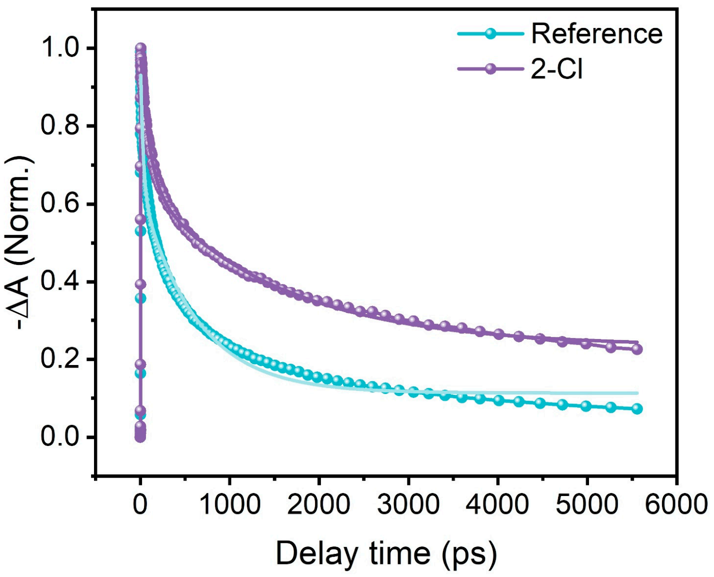

Figure S6. The normalized fs-TA kinetics of perovskite films without and with 2-Cl addition.

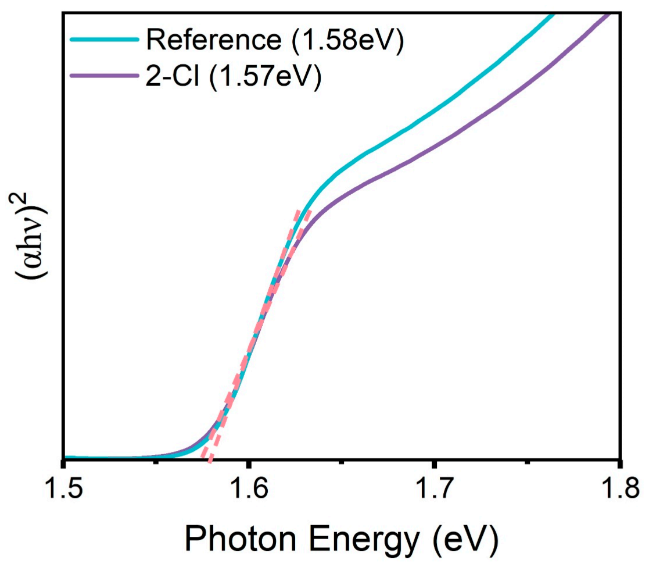

Figure S7. Tauc plots of perovskite films extracted from the UV-Vis absorption spectra.

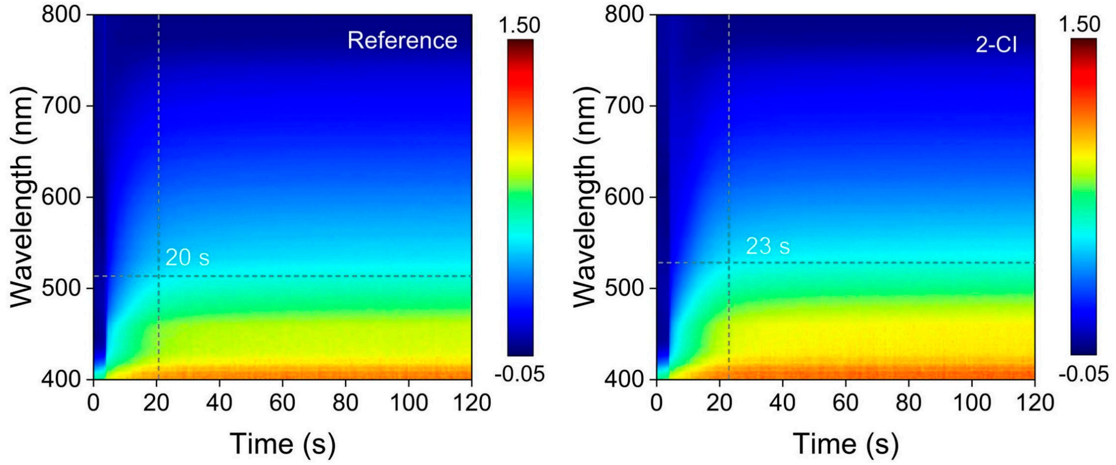

Figure S8. In-situ UV-Vis absorption mapping of perovskite films.

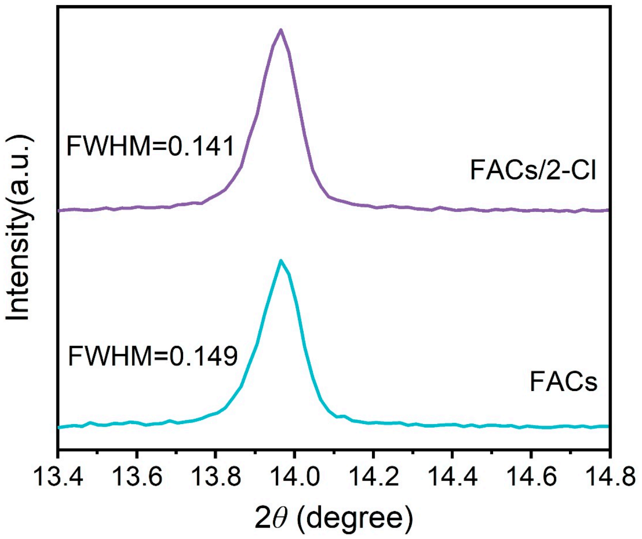

Figure S9. Full width at half maximum (FWHM) of (001) peaks calculated by the XRD results of the reference and 2-Cl perovskite films.

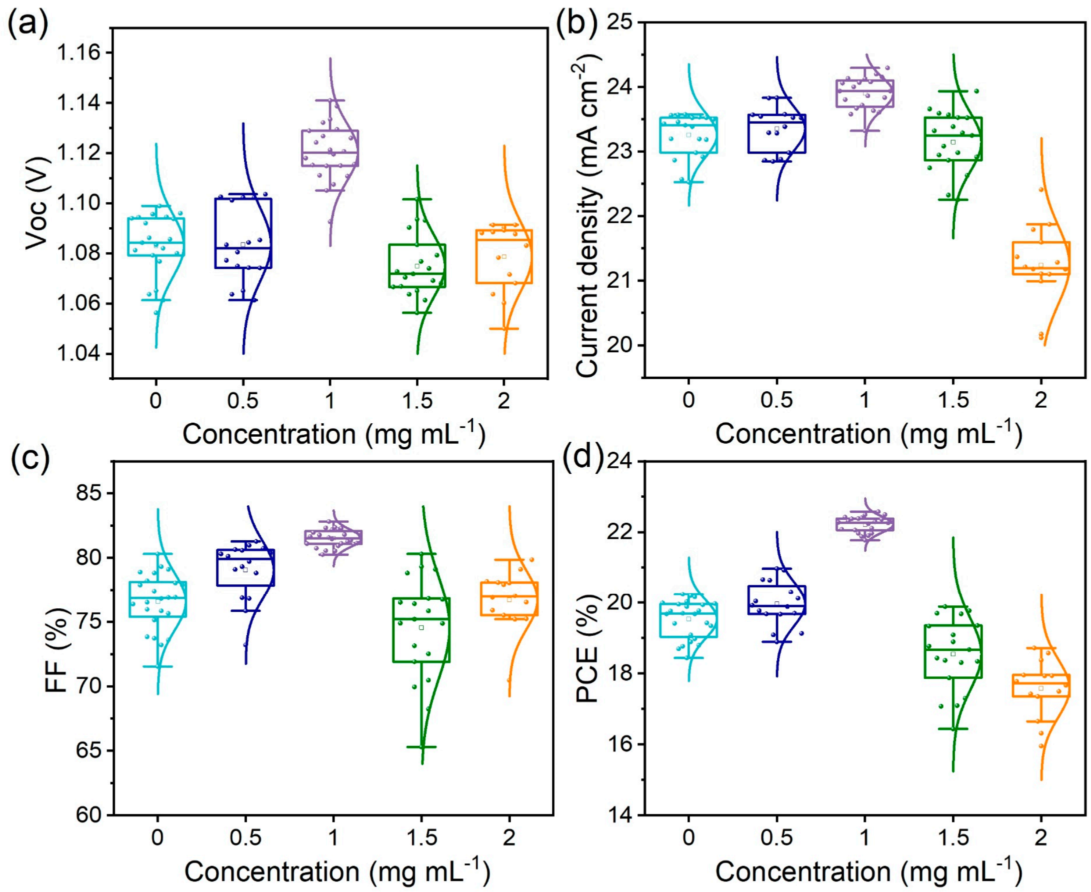

Figure S10. The impact of 2-Cl concentration on device photovoltaic performance.

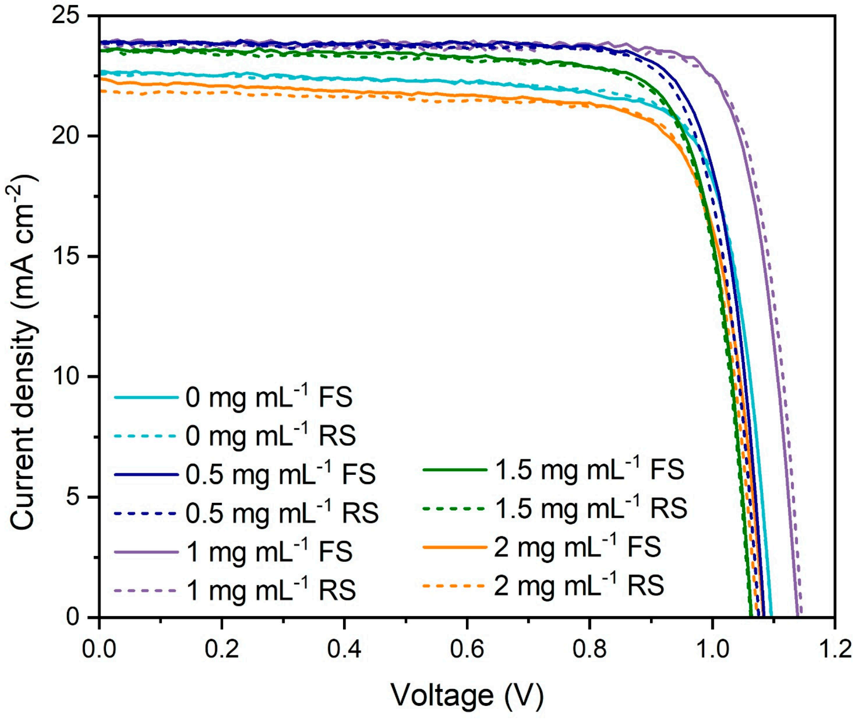

Figure S11. *J*−*V* curves of the optimal devices doped with 2-Cl at various concentrations.

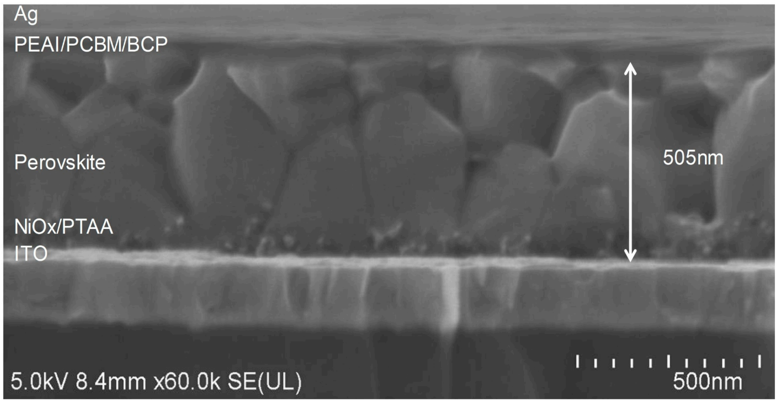

Figure S12. Cross-sectional SEM image of the fabricated device with 2-Cl.

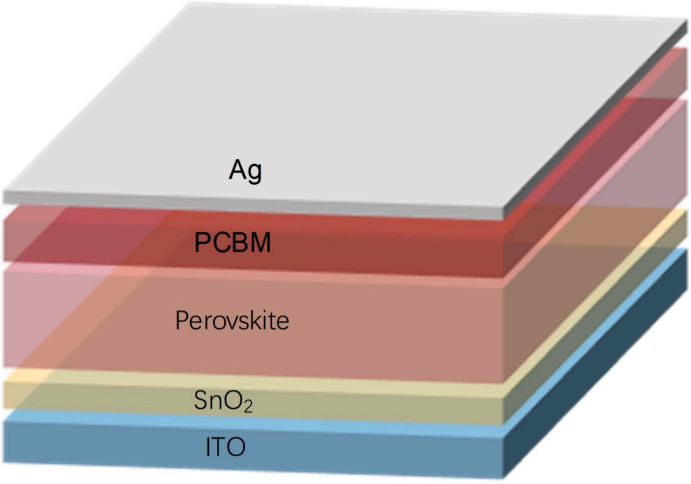

Figure S13. Structure of the electron-only device used for SCLC measurements.

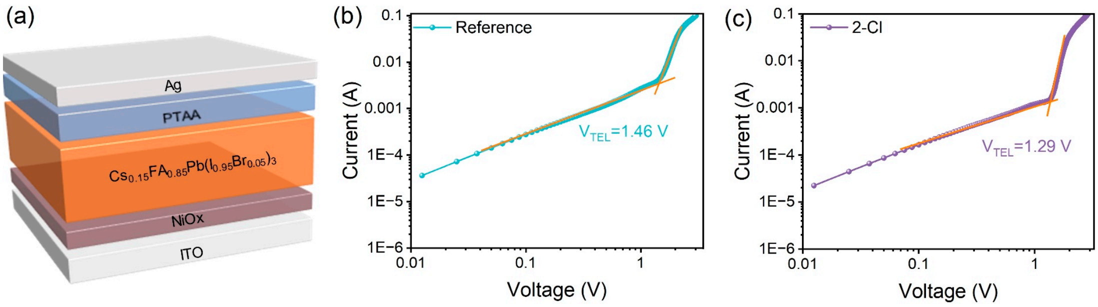

Figure S14. Structure and results of the hole-only device used for SCLC measurements.

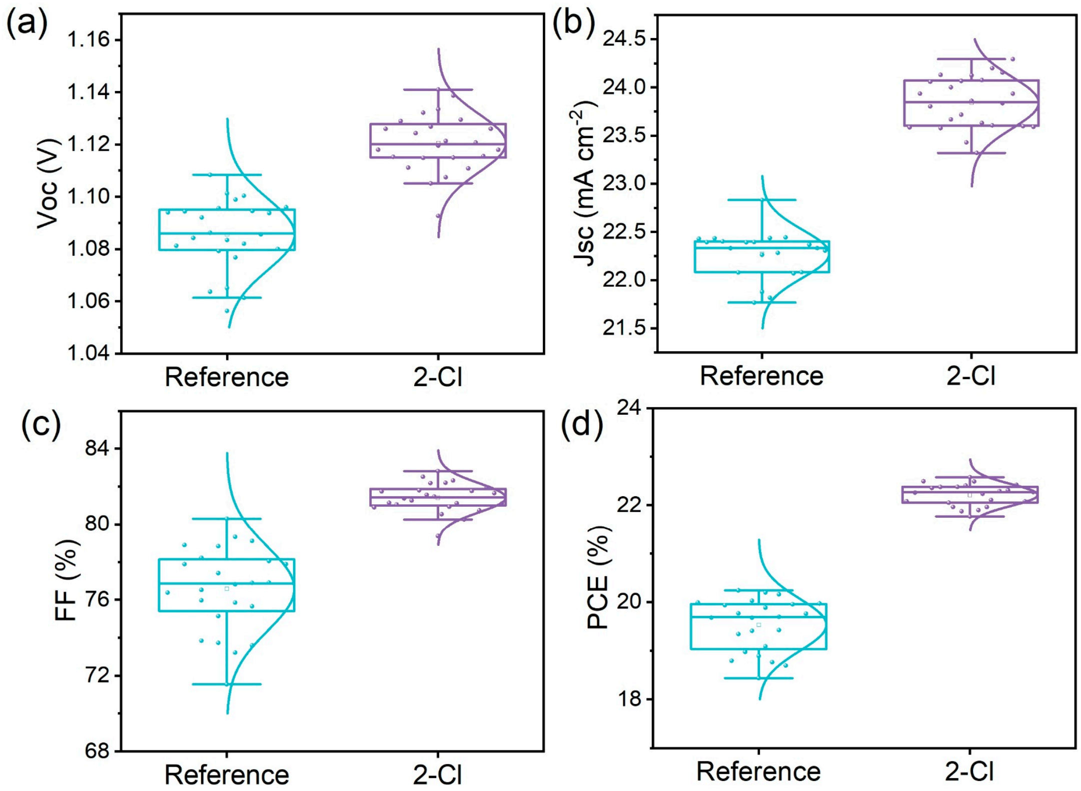

Figure S15. Statistic photovoltaic parameters of PSCs based on the reference and 2-Cl-modified perovskite films.

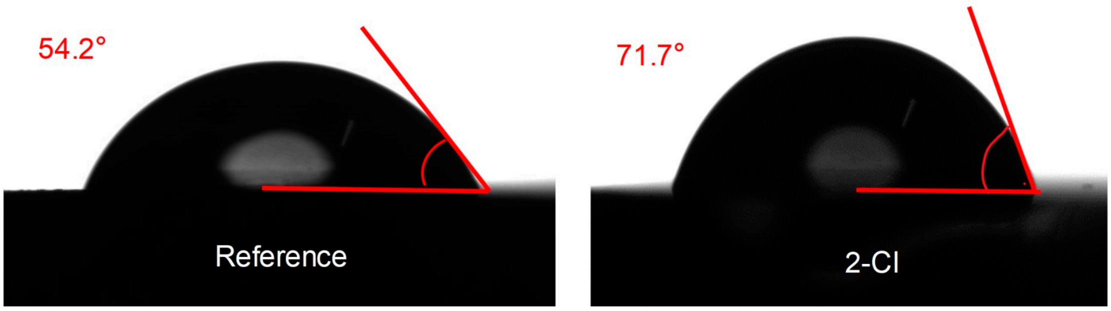

Figure S16. Contact angle results of perovskite films without (reference) and with 2-Cl.

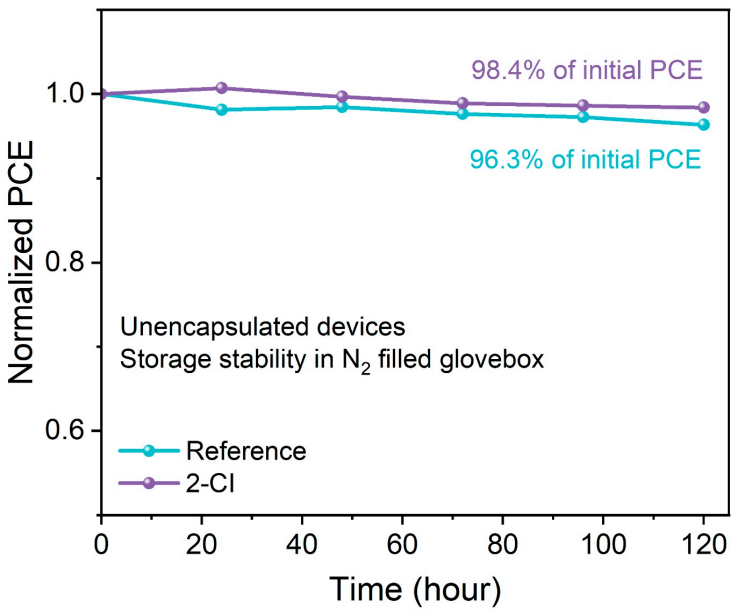

Figure S17. Storage stability of the fabricated devices in a N_2-filled glovebox.

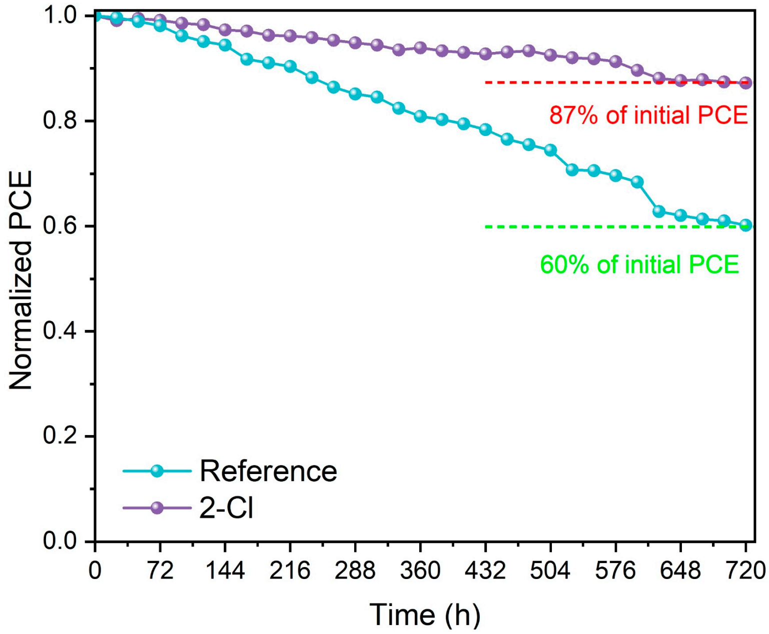

Figure S18. Thermal stability of the reference and 2-Cl-modified devices.

Table S1. Electronic parameters of the reference and 2-Cl passivated perovskite films derived from the UPS results.

Sample	E_{cutoff} (eV)	E_{onset} (eV)	VB (eV)	E_g (eV)	CB (eV)
Reference	16.44	0.99	-5.77	1.58	-4.19
2-Cl	16.48	1.05	-5.79	1.57	-4.22

Table S2. Summary of TRPL lifetimes for the reference and 2-Cl-passivated perovskite films with a concentration of 1 mg mL^{-1}.

Samples	A_1	τ_1 (ns)	A_2	τ_2 (ns)	τ_{ave} (ns)
Reference	0.46	44.34	0.50	403.37	370.40
2-Cl	0.34	7.60	0.65	807.29	812.03

Table S3. Parameters of 2-Cl-doped devices with various concentrations.

Concentrations	Champion/average	J_{SC} (mA cm^{-2})	V_{OC} (V)	FF (%)	PCE (%)
0 mg mL^{-1}	Champion	23.43	1.09	78.89	20.24
	Average	23.42±0.56	1.08±0.03	75.92±4.38	18.34±0.90
0.5 mg mL^{-1}	Champion	23.88	1.08	80.97	20.97
	Average	23.59±0.73	1.07±0.02	77.25±4.04	19.93±1.04
1 mg mL^{-1}	Champion	23.94	1.14	82.82	22.58
	Average	23.81±0.14	1.12±0.02	81.54±1.28	22.18±0.40
1.5 mg mL^{-1}	Champion	23.57	1.06	79.32	19.89
	Average	23.09±0.84	1.08±0.02	72.80±7.50	18.17±1.73
2 mg mL^{-1}	Champion	21.87	1.07	79.86	18.72
	Average	21.03±0.91	1.07±0.02	71.20±8.67	16.41±1.79

Table S4. Fitting parameters of impedance spectroscopy for the reference and 2-Cl-doped devices.

Sample	R_S (Ω)	R_{rec} (Ω)
Reference	12.45	678.00
2-Cl	9.81	1696.00

Table S5. Photovoltaic parameters of the reference and 2-Cl-doped devices at a concentration of 1 mg mL^{-1}.

Samples	Champion/average	J_{SC} (mA cm^{-2})	V_{OC} (V)	FF (%)	PCE (%)
Reference	Champion	23.43	1.09	78.89	20.24
	Average	23.42±0.56	1.08±0.03	75.92±4.38	18.34±0.90
2-Cl	Champion	23.94	1.14	82.82	22.58
	Average	23.81±0.14	1.12±0.02	81.54±1.28	22.18±0.40
